# Comparative Responses of Orange-Foot and Common-Foot *Haliotis gigantea* to Carotenoid-Enriched Diets: Survival, Heat Tolerance, and Bacterial Resistance

**DOI:** 10.3390/ani14020180

**Published:** 2024-01-05

**Authors:** Yizhou Ke, Shuyi Liu, Wencui Zeng, Xiaolong Gao, Mingyi Cai, Weiwei You

**Affiliations:** 1 Key Laboratory of Healthy Mariculture for the East China Sea, Ministry of Agriculture and Rural Affairs, Jimei University, Xiamen 361021, China; lsy00410@163.com (S.L.); myicai@jmu.edu.cn (M.C.); 2 State Key Laboratory of Mariculture Breeding, Fisheries College, Jimei University, Xiamen 361021, China; 3 State Key Laboratory of Mariculture Breeding, College of Ocean and Earth Sciences, Xiamen University, Xiamen 361102, China; kyz2010@126.com (W.Z.); xlgao@xmu.edu.cn (X.G.); 4 Fujian Key Laboratory of Genetics and Breeding of Marine Organisms, Xiamen University, Xiamen 361102, China; 5 National Observation and Research Station for the Taiwan Strait Marine Ecosystem, Zhangzhou 363400, China

**Keywords:** *Haliotis gigantea*, carotenoids, survival rate, heat tolerance, bacterial resistance

## Abstract

**Simple Summary:**

This study explored how adding carotenoids, natural substances known for boosting survival, heat resistance, and immunity against bacteria, affects the abalone species *Haliotis gigantea*. We tested 13 different types of feed, each with varying amounts of three carotenoids: astaxanthin, zeaxanthin, and *β*-carotene. These feeds were given to two types of abalones: those with common (pale yellow) and orange-colored foot muscles. The study found that all abalones, regardless of the carotenoid levels in their diet, had a survival rate of about 70–80%. This suggests that these added nutrients do not significantly change their survival chances. Interestingly, when exposed to heat, the common-footed abalones were able to stay attached longer than the orange-footed ones, indicating that they might benefit more from the added nutrients. In addition, both types showed similar resistance to bacterial infection. Our findings highlight the potential of using carotenoid-enriched diets in aquaculture to improve the health and resilience of abalones, offering insights for developing better feeds for these valuable sea creatures.

**Abstract:**

Carotenoids, known to enhance survival, heat tolerance, and bacterial resistance, play an essential role in the nutrition of economically important aquatic animals. This study specifically examined their impact as feed additives on the abalone *Haliotis gigantea*. We prepared 13 compound feeds with varying levels of astaxanthin, zeaxanthin, and *β*-carotene, and administered them to both common-footed and orange-footed *H. gigantea*. The survival rate of *H. gigantea* was about 70–80%, with no significant differences in survival observed among the various carotenoid-supplemented feeding groups or when compared with the control group, nor between orange-footed and common-footed individuals. In heat attachment duration experiments, orange-foot abalones exhibited longer attachment durations with certain concentrations of astaxanthin and zeaxanthin, whereas common-foot abalones showed extended durations with astaxanthin, zeaxanthin, and *β*-carotene, indicating that common-foot abalones might benefit more from these carotenoids. Additionally, our results showed similar patterns and levels of *Vibrio harveyi* AP37 resistance in both orange-footed and common-footed *H. gigantea*, suggesting a uniform response to carotenoid supplementation in their bacterial defense mechanisms. This study suggests the potential benefits of carotenoid supplementation in *H. gigantea* and contributes to the theoretical basis for developing high-quality artificial compound feeds.

## 1. Introduction

Carotenoids are pivotal pigments for aquatic animals, playing crucial roles in their coloration, growth, and reproduction [1]. These animals cannot synthesize carotenoids, requiring them to obtain these essential compounds from their diet. Carotenoids are extensively used as coloring agents and feed additives in aquaculture, providing significant health benefits to aquatic animals [2,3,4]. Astaxanthin, zeaxanthin, and *β*-carotene, often hailed as advanced food and animal feed additives in the market, showcase remarkable antioxidant activities. These additives confer significant health benefits not only to animals but also to humans [5,6]. These compounds fortify the immune system, offer protection from sunburn, and may even play roles in countering obesity [7]. Hence, consistent intake of seafood rich in carotenoids is essential for enhancing health and preventing diseases.

*Haliotis gigantea*, a predominant economic abalone species, thrives along the coastlines of Asian countries [8]. While the majority exhibit pale yellow foot muscles, a rare mutant variant with orange foot muscles exists, referred to as orange-footed *H. gigantea*. The carotenoid content in these orange-footed abalones reaches a remarkable 300–500 μg/dry g [9], making it the highest among shellfish to date. This is approximately 16.5 times more than their common-footed counterparts and over 100 times more than most other edible shellfish [10]. Their vibrant coloration and high carotenoid content position them favorably in premium markets. Beyond genetic predispositions, dietary carotenoids play a significant role in manifesting the orange-foot trait in *H. gigantea*. Artificial compound feeds, as opposed to large algae, often promote better growth in *H. gigantea* [11]. In regions with a dearth of large algae, the potential for raising abalones on these feeds is promising. Although past research has highlighted the influence of dietary carotenoids on the coloration of abalone foot muscles [6], there is limited investigation into their impact on the resistance between orange-footed and common-foot *H. gigantea*.

Temperature, as one of the paramount environmental factors in aquaculture, plays a crucial role in abalone growth. Optimal temperatures not only maximize abalone production but also minimize mass mortality during cultivation [12]. Yet, with the global rise in temperatures due to climate change, the abalone industry faces challenges with implications for the growth, health, and survival of these mollusks [13]. Carotenoids serve as potent antioxidants in aquatic creatures, bolstering their resistance to temperature fluctuations [14].In addition, with an increasingly challenging environment and rising temperatures [15], an abalone’s heat tolerance becomes a vital metric in its farming. The recent introduction of the heat adhesion duration (HAD) method, which measures abalone adhesive capacity, offers a high-throughput evaluation [16,17,18].

The expansion of abalone farming, combined with overstocking and heightened offshore pollution in recent years, has heightened the vulnerability of abalones to bacterial threats, leading to significant die-offs [19,20]. A primary culprit is *Vibrio harveyi*, a pathogenic bacterium causing considerable abalone mortality [21]. Outbreaks of this bacterium have caused extensive losses in abalone populations, translating to substantial economic setbacks [22]. Carotenoids, with antioxidant properties surpassing vitamin E, play a crucial role in preventing cell damage from free radicals and have been incorporated as additives in aquaculture. Fish rich in carotenoids, for instance, display heightened resistance against bacterial and fungal threats and exhibit enhanced bacterial resistance [23,24].

In this study, we investigated the impact of thirteen different feed formulations on *H. gigantea*, each enriched with varying concentrations of three carotenoids: astaxanthin, zeaxanthin, and *β*-carotene. The primary objective was to evaluate the effectiveness of these carotenoid-enriched diets in improving the survival rates, thermal tolerance, and bacterial resistance of *H. gigantea*. This research was aimed at providing an understanding of how these dietary variations affect both common-foot and orange-foot variants of *H. gigantea*, offering valuable insights for enhancing seafood production and abalone aquaculture practices.

## 2. Materials and Methods

### 2.1. Experiment Diet and Analysis

**Figure 1:**
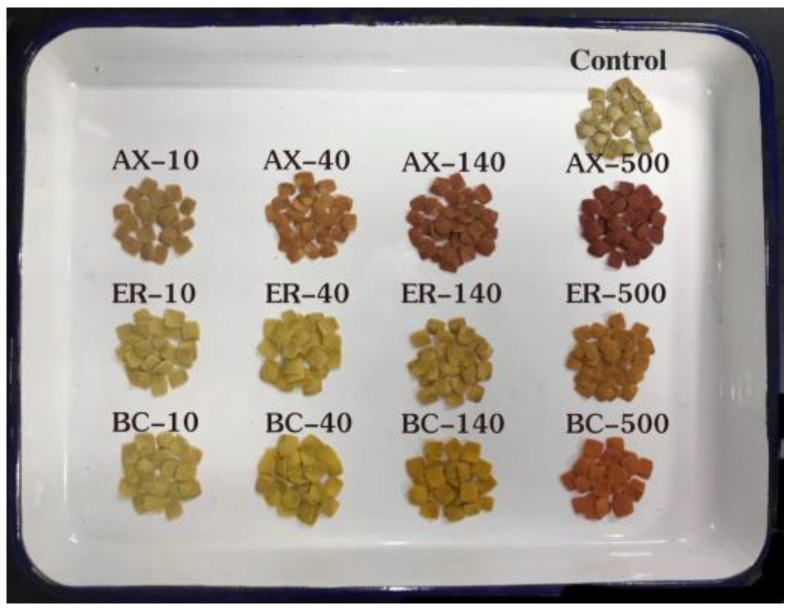
Visual representation of 13 different feed types.

Thirteen compound feeds (Figure 1) were prepared using the formulation detailed in Table 1. As contained in these compound feeds, the protein sources were casein and bean pulp, the fat sources were soybean oil and perilla oil, the carbohydrate sources were bean pulp, α-starch, flour, and sodium fucoidan, and the binders were α-starch, sodium fucoidan, and sodium carboxymethyl cellulose. The materials were added to the mixer and blended well; pure water equivalent to 30% of the gross weight of the materials was added, stirred until flocculent was formed, and then poured into a low-temperature puffed feed press to make flake feeds of 1 cm × 1 cm × 1 mm. Dried in a constant temperature oven at 55 °C for 12 h, cooled, and stored in a −20 °C refrigerator under shaded conditions. For the main nutritional components contained in the compound feeds, refer to Table 2. We measured the relative content of moisture, ash, crude protein, crude fat, and carbohydrates in each type of feed. Detailed measurement methods are referred to in the Supplementary Materials.

### 2.2. Determination of Carotenoid Content in the Feed

The determination of carotenoid content was based on methodologies from previous literature [9]. Briefly, tissue specimens underwent vacuum freeze drying for 48 h and were then ground to a powder. A total of 1.00 g of this powder was placed in a centrifuge tube with 20 mL of ethyl acetate and 0.02 g of BHT. The mixture was homogenized and centrifuged to separate the supernatant, repeating this three times. All supernatants were evaporated in a flask, and then the carotenoids were transferred to a 4 mL vial and concentrated. Before analysis, the extracts were dissolved in ethyl acetate and treated with ultrasound, then filtered and prepared for HPLC.

The mobile phase was n-hexane and ethyl acetate (40:60 *v*/*v*), flow rate of 1.5 mL/min, and used a YMC-PackSIL column at 25 °C with Chromeleon Console software (Version 7.2.10) for data analysis. Astaxanthin, zeaxanthin, and *β*-carotene standards were dissolved in ethyl acetate and transferred into volumetric flasks. After dilution, standard solutions were analyzed chromatographically to calculate standard curves. Using similar conditions, the sample solution was analyzed, and carotenoids in the specimens were identified. The content of astaxanthin, zeaxanthin, and *β*-carotene was determined using the standard curve. For the content of carotenoids, refer to Table 3.

### 2.3. Feeding Trial and Culture Management

The materials intended for the experiment were sourced from the 6-month-old *H. gigantea* population bred from Fuda abalone farm in mid-November 2019. The initial shell length of *H. gigantea* is referred to in Table S1. We selected 6000 healthy and uniformly sized *H. gigantea*, with half being orange-footed and the other half being common-footed. For clarity in our experiment, the orange-footed individuals were labeled as “RR” and the common-footed as “SS”. Based on feed type, they were categorized into 13 groups: control, AX-10, AX-40, AX-140, AX-500, ER-10, ER-40, ER-140, ER-500, BC-10, BC-40, BC-140, BC-500. The naming convention for the feed is “carotenoid type + carotenoid concentration”. Specifically, AX, ER, and BC stand for astaxanthin, zeaxanthin, and *β*-carotene, respectively. For instance, “AX-10” indicates that the feed contains 10 mg/kg of astaxanthin. Further, based on their type, they were divided into 26 subgroups: orange-footed (RR) and common-footed (SS). Each abalone’s shell was marked accordingly before the study began.

After labeling, all abalones underwent a 7-day acclimation in the experimental setup to offset any artificial disturbance. Abalones were randomly distributed across 39 net cages (65 cm × 35 cm × 70 cm), each consisting of an outer dark green plastic mesh and a perforated corrugated board. Each cage housed 75 SS and 75 RR abalones. Every cage received a set feed amount until satiation. All treatments had three replicates, and except for the feed type, culture conditions, including maintaining a temperature range of 14–20 °C, remained consistent. The 130-day experiment involved feeding at 5:00 p.m. daily, with leftover feed cleared, deceased abalones removed, and cages cleaned by 9:00 a.m. the following day.

### 2.4. Determination of Survival Rate

Throughout the cultural period, daily mortality was recorded. Deceased abalones were promptly cleared and cages cleaned. Post experiment, survival rates for both common-footed and orange-footed abalones were computed and analyzed using the following equation:

$$\mathrm{Survival}\;\mathrm{rate}\left(\%\right)=(\frac{number\;of\;surviving\;individuals\;after\;the\;experiment}{total\;number\;of\;individuals\;before\;the\;experiment})\times 100$$

### 2.5. Thermal Tolerance Assessment

The abalones used for the experiment were *H. gigantea* cultured for 120 days, as described in Section 2.2. The heat attachment duration (HAD) experiment began by selecting 9 abalones from each group. Their shell labels were recorded before they were moved to a 20 °C recirculating system for a 5-day acclimation. Throughout this period, abalones from the same group were distributed across four tanks to minimize environmental variances. After acclimation, most abalones attached inside a circular substrate. This substrate was then elevated above the water, and any remaining abalones were carefully moved inside it. The water temperature was then raised to 32 °C at a rate of 1 °C per hour. Once this temperature was achieved, observations began. Every 5 min, falling abalones were noted, recording their attachment time and label numbers.

### 2.6. Bacterial Challenges

#### 2.6.1. Grouping of Abalones

The abalones used for the experiment were *H. gigantea*, cultured for 120 days, as detailed in Section 2.2. We chose a concentration of 500 µg/g for each carotenoid type. They were categorized into four groups: AX, ER, BC, and CON. AX, ER, BC, and CON stand for astaxanthin, zeaxanthin, *β*-carotene, and control, respectively. Additionally, the orange-foot and common-foot abalones were denoted as RR and SS, respectively. This led to the formation of eight distinct groups: AX-RR, AX-SS, ER-RR, ER-SS, BC-RR, BC-SS, CON-RR, and CON-SS.

#### 2.6.2. Preparation of Bacterial Solution

From a −80 °C freezer, *Vibrio harveyi* AP37 samples were carefully thawed on ice. In a sterile environment, the activated bacterial solution’s supernatant was spread onto a 2216E agar plate and incubated at 28 °C for a day. *V. harveyi* AP37, extracted from diseased *Haliotis diversicolor*, exhibits pathogenic effects on abalone, as identified by a previous study [25]. Next, single colonies were picked and cultured again on fresh plates. These isolated colonies were inoculated into 3 L of 2216E liquid broth and agitated at 180 rpm at 28 °C until the bacterial concentration reached approximately 3 × 10^9^ cfu/mL. This culture was then distributed into 60 centrifuge tubes, each holding about 3 × 10^9^ cfu/mL. The bacterial count in our bacterial solutions was measured using flow cytometry (CytoFLEX, Beckman Coulter, Brea, CA, USA). After centrifuging at 4000 rpm for 10 min, the clear supernatant was discarded, and the remaining bacterial pellet was reconstituted in 50 mL of sterile seawater. This freshly prepared bacterial solution was used immediately for experiments.

#### 2.6.3. Assessment of Bacterial Resistance of *H. gigantea*

From each group, 30 specimens were allocated to 12 tanks, each holding 30 L of water infused with a *V. harveyi* suspension at a concentration of 1 × 10^7^ cfu/mL. Additionally, 20 specimens from each group were placed into three similarly sized tanks filled with regular seawater, serving as the control. Throughout the 72 h study, the water in each tank was fully replaced at 9:00 a.m. daily, with fresh bacterial solution added to maintain the *V. harveyi* concentration at 1 × 10^7^ cfu/mL. To collect blood samples from the abalones, we made a small incision near the heart region along the midline of the pedal (foot) area, where the blue blood typically flows out. We then rapidly collected the blood using a pipette and transferred it into centrifuge tubes, which were immediately stored in liquid nitrogen. Blood samples were drawn from specimens at specified intervals (0, 6, 12, 24, 48, and 72 h) during the challenge test and compared with the 0 h and 72 h samples from the control group. These samples were centrifuged at 1000 rpm for 3 min to obtain the serum.

For the various assessments, the following kits were utilized: The “Total Antioxidant Capacity Assay Kit (S0119) with a Rapid ABTS method” from Beyotime Biotechnology, Shanghai, China, was used to measure total antioxidant capacity (TAC). The “CuZn/Mn-SOD activity assay kit (S0101S, WST-8 method)”, also by Beyotime Biotechnology, China, was employed for evaluating superoxide dismutase (SOD) activity. Additionally, the “Malonaldehyde (MDA) assay kit (A003-1-1, TBA method)” sourced from Nanjing Jiancheng Bioengineering Institute, Nanjing, China, was used to determine the malondialdehyde (MDA) content [26].

### 2.7. Data Analysis

We applied a one-way ANOVA to assess mean discrepancies among the groups. If notable differences were detected (*p* < 0.05), a follow-up Tukey post hoc analysis was performed to isolate the causes of such variations. All statistical analyses were carried out using IBM SPSS Statistics 26, and graphs were plotted in GraphPad Prism 8.0. Data contained in all graphs are expressed as mean ± standard deviation.

## 3. Results

### 3.1. Survival Rate of Abalones

**Figure 2:**
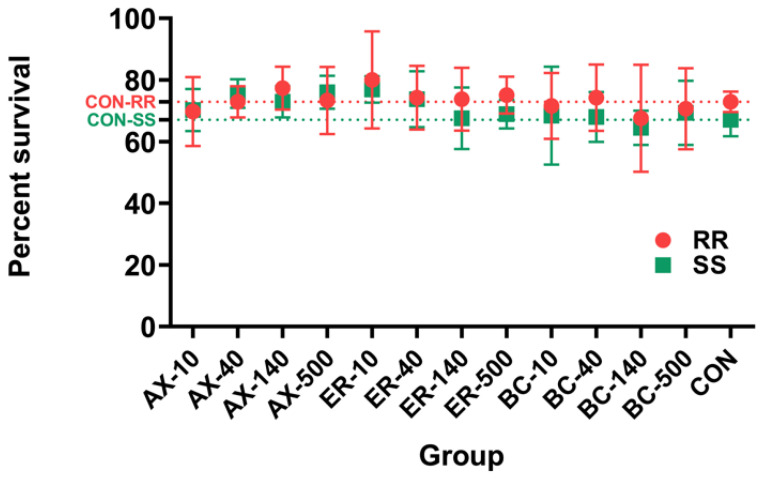
Comparison of the survival rate using different abalone diets. RR and SS stand for orange-foot and common-foot abalone, respectively.

Figure 2 depicts the survival rate of abalone after 130 days of feeding experiment, which varied between approximately 70% and 80%. No differences among different feeding groups or within the same group were observed (*p* > 0.05). Within the same group, orange-footed individuals exhibited a slightly higher survival rate compared with their common-footed counterparts, though this difference was not statistically significant (*p* > 0.05).

### 3.2. HAD Comparison of Abalones across the Diet Groups

**Figure 3:**
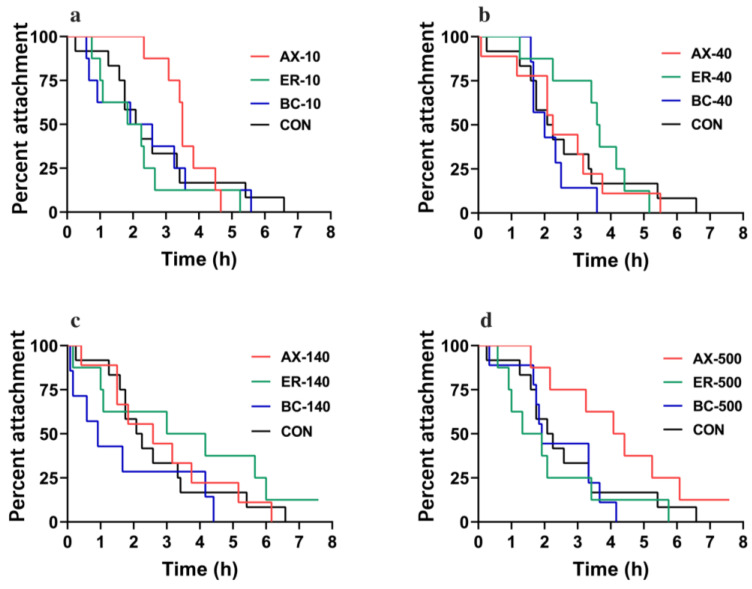
Comparison of heat adhesion duration (HAD) trial of orange-foot abalone at 10 g/kg (**a**), 40 g/kg (**b**), 140 g/kg (**c**), and 500 g/kg (**d**) carotenoid concentration in feeds. AX, ER, BC, and CON stand for astaxanthin, zeaxanthin, *β*-carotene, and control, respectively. Each group contained nine individuals.

**Figure 4:**
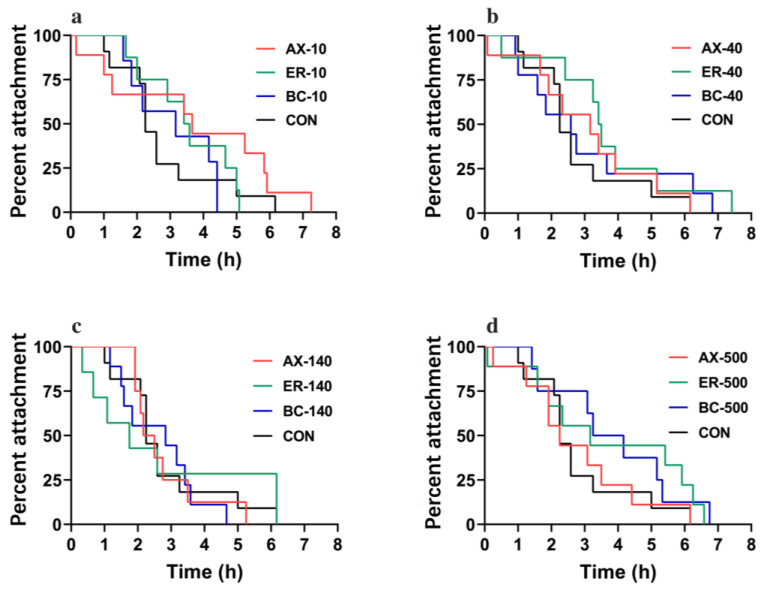
Comparison of heat adhesion duration (HAD) trial of common-foot abalone at 10 g/kg (**a**), 40 g/kg (**b**), 140 g/kg (**c**), and 500 g/kg (**d**) carotenoid concentration in feeds. AX, ER, BC, and CON stand for astaxanthin, zeaxanthin, *β*-carotene, and control, respectively. Each group contained nine individuals.

Figure 3 and Figure 4 show the variations in the HAD trial of abalones at different concentrations and different types of feeds. Across all concentrations, a gradual decline in percent attachment over time was observed, with no group maintaining complete adherence after 8 h. For orange-foot abalone individuals, the median attachment duration (HAD_50_) of ER-40, AX-10, and AX-500 groups was 3.61, 3.54, and 4.22 h, respectively, which was higher than the control group (HAD_50_ = 2.32 h) (Figure 3), while the HAD_50_ in other carotenoid concentrations in feeds had no significant differences with the control group. For common-foot abalones, in general, adding astaxanthin, zeaxanthin, and *β*-carotene as additives in feeds increased the attachment duration for most concentration sets (Figure 4).

### 3.3. Variations in Biochemical and Antioxidant Parameters across the Diet Groups

**Figure 5:**
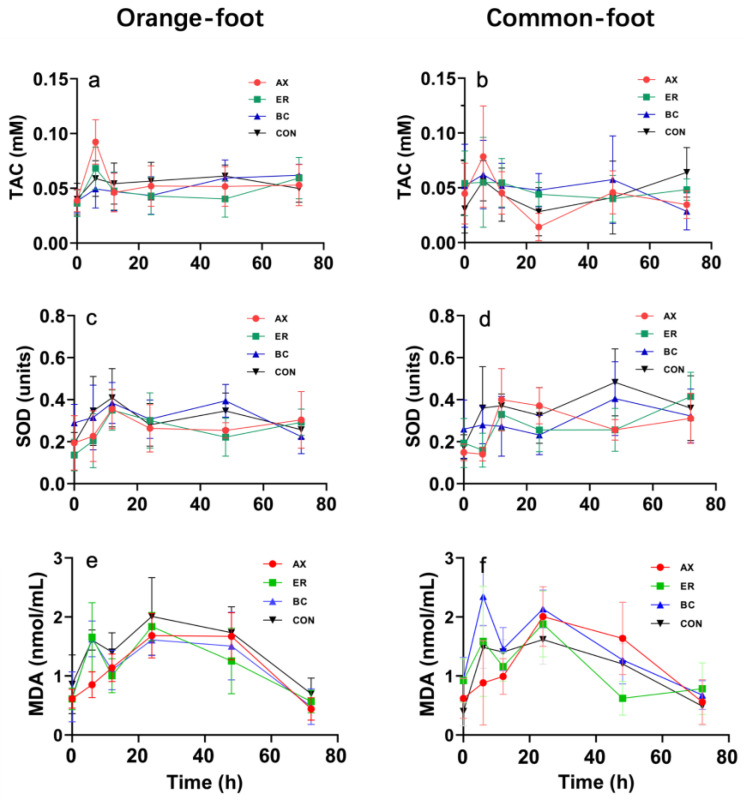
Biochemical and antioxidant enzymes in the serum of *H. gigantea* fed diets with graded levels of carotenoid additives for 72 h. Subfigures (**a**–**f**) show the levels of specific enzymes and compounds: (**a**,**b**) total antioxidant capacity (TAC), (**c**,**d**) superoxide dismutase (SOD) activity, and (**e**,**f**) malondialdehyde (MDA) content in the serum of *H. gigantea.* AX, ER, BC, and CON stand for astaxanthin, zeaxanthin, *β*-carotene, and control, respectively. Error bars represent standard deviations from the mean (*n* = 3).

Figure 5 shows the temporal variations in TAC, SOD activity, and MDA levels over a 72 h period after administering diets with graded levels of carotenoid additives to both orange-foot and common-foot abalones. TAC, SOD, and MDA exhibited comparable expression levels in both orange-foot and common-foot abalone types after the challenge, with no discernible differences between them.

Both abalone types demonstrated a peak in TAC at the 6 h mark postchallenge, subsequently declining and stabilizing thereafter. Notably, the TAC values for both the AX and ER groups in both abalone types surpassed that of the control group at the 6 h interval. Conversely, the TAC value for the BC group was observed to be inferior to the control group at this same time point.

For orange-foot abalones, the trajectory of SOD activities largely paralleled that of TAC. Specifically, SOD activities for the AX and ER groups reached their zenith at 12 h post-challenge before plateauing. Both the BC and CON groups, however, exhibited dual peaks at 12 and 48 h. Both the AX and ER groups of common-foot abalones demonstrated an increase followed by a subsequent decrease, implying a singular peak at 12 h. Notably, only the AX group exhibited SOD activities surpassing the control group between the 12 to 24 h interval.

MDA content demonstrated two successive cycles of increase followed by a decrease. For both the orange-foot and common-foot abalones within the AX group, a singular peak was discerned between 24 to 48 h. In contrast, other abalone groups registered dual peaks at 6 and 24 h. For the common-foot abalones, the MDA levels in the BC group consistently exceeded those in the control group.

## 4. Discussion

Based on our results, there was no difference in survival among *H. gigantea* groups, suggesting that introducing astaxanthin, zeaxanthin, and *β*-carotene to their diet does not significantly affect their survival rate. The similar survival rates observed between orange-foot and common-foot abalones, even with the naturally higher carotenoid content in orange-foot variants, indicate that the variations in carotenoid levels might not substantially influence the abalones’ intrinsic resilience or tolerance mechanisms. This observation could be attributed to the culture conditions, especially the temperatures, which were maintained within the optimal, stress-free range for abalone. Meng et al. [14] demonstrated that as the temperature rose from 26 °C to 34 °C, the survival rate of *Pinctada fucata* significantly declined. Additionally, a higher total carotenoid content (TCC) corresponded to a greater survival rate, highlighting the positive relationship between survival rate and TCC levels. In related studies on other aquatic species, Harpaz et al. [27] noted that while adding carotenoids to the *Cherax quadricarinatus* diet enhanced its body color, it did not impact its growth or survival. Our findings align with these observations. However, another case indicated that adding *β*-carotene to the diet for abalone *Haliotis discus hannai* can help improve the survival rate [28], highlighting the potential for varying responses within different aquatic populations. This variability calls for a more nuanced approach to dietary supplementation in aquaculture, taking into account the unique biological and environmental needs of each species.

Abalone has to experience metamorphosis in the growth and development stage and use their gastropods to firmly attach to the surface of an object after the metamorphosis process [29]. In this sense, attachment is one of the key measures of an abalone’s viability. HAD serves as a reliable measure of resistance, which is invariant to the abalone’s size, age, or gender [18]. Previous studies indicated that the addition of carotenoids to the diet would improve the resistance of mollusks under heat stress. Ma et al. [30] reported that the addition of 80 mg/kg astaxanthin can reduce the detachment and mortality of *H. discus hannai* under 30 °C of heat stress, which is broadly in line with the results reported in our study. Our results also indicate that the inclusion of carotenoids in the diet extends the attachment duration for common-foot abalones, but not for orange-foot individuals in most cases. A possible explanation is that orange-foot individuals might already have higher baseline levels of certain carotenoids due to their genetic makeup or inherent dietary habits. As a result, additional supplementation might not cause a noticeable change in their attachment behavior, whereas the common-foot individuals, having a different carotenoid profile, might benefit more evidently from the supplementation.

The abalone’s immune system is known to respond to bacterial and viral threats [31,32]. For instance, a study giving intramuscular injection of viral hemorrhagic septicemia virus (VHSV) to abalone *H. discus hannai*, with the injection of phosphate-buffered saline (PBS) as the control group, the mRNA expression levels of the superoxide dismutase of the infected abalone gradually increased and peaked at 12 h, and its findings suggest that VHSV infection can induce antioxidant enzymes in abalone, implying the possible role of abalone in activating innate immune defense responses against infection [33]. In our study, in general, *H. gigantea*’s immune parameters responded similarly to the *V. harveyi* bacterial challenge, revealing analogous antioxidant response patterns in both orange-foot and common-foot abalones. The orange-foot and common-foot variants of *H. gigantea*, while distinguishable, likely share a significant amount of genetic material that governs their immune response. This shared genetic basis could result in similar immune system functioning and antioxidant responses. Carotenoids, as known antioxidants, may bolster the organism’s ability to mitigate oxidative stress [34]. Meng et al. [14] found that when the total carotenoid content of *P. fucata* was higher, the levels of SOD and MDA tended to be lower, suggesting a potential protective role of carotenoids in *P. fucata*. A possible explanation is that elevated total carotenoid content could enhance the organism’s capacity to neutralize reactive oxygen species (ROS), which, in turn, might reduce the need for high SOD activity. Our findings in *H. gigantea* do not entirely align with these observations. This variation could be due to species-specific differences in how carotenoids are metabolized and utilized by different types of abalone.

## 5. Conclusions

This study’s investigation into the effects of carotenoid supplementation on *H. gigantea* has led to several conclusions. Firstly, carotenoid-enriched diets showed no significant differences in survival rates among the various carotenoid-supplemented feeding groups compared with the control group or between the orange-footed and common-footed variants. This suggests that while carotenoids are beneficial in other aspects, they do not drastically alter the survival rates of these abalones. Secondly, the heat attachment duration experiments revealed that common-foot abalones may derive more pronounced benefits from specific types of carotenoids, such as astaxanthin and zeaxanthin, compared with orange-foot abalones. This suggests a potential variance in how different abalone types respond to carotenoid types. Additionally, the study found that both orange-footed and common-footed *H. gigantea* exhibit similar patterns and levels of pathogen resistance, indicating a generally uniform response to carotenoid supplementation in their bacterial defense mechanisms across different abalone types. These findings enhance our understanding of carotenoid supplementation’s role in aquaculture nutrition and support the development of high-quality, species-specific artificial compound feeds for *H. gigantea*, potentially improving their health and resilience in aquaculture settings.

## Figures and Tables

**Table 1:** Formulation and proximate composition of the experimental diets, percentage dry weight basis (dry matter basis, g/kg).

Ingredient	Experimental Diet												
	Control	AX-10	AX-40	AX-140	AX-500	ER-10	ER-40	ER-140	ER-500	BC-10	BC-40	BC-140	BC-500
Casein	180	180	180	180	180	180	180	180	180	180	180	180	180
Soybean meal	245	245	245	245	245	245	245	245	245	245	245	245	245
α-Amylase	120	120	120	120	120	120	120	120	120	120	120	120	120
Flour	140	140	140	140	140	140	140	140	140	140	140	140	140
Soybean oil + Perilla seed oil	40	40	40	40	40	40	40	40	40	40	40	40	40
Vitamin premix ^a^	20	20	20	20	20	20	20	20	20	20	20	20	20
Mineral premix ^b^	40	40	40	40	40	40	40	40	40	40	40	40	40
Sodium alginate	150	150	150	150	150	150	150	150	150	150	150	150	150
Choline chloride	5	5	5	5	5	5	5	5	5	5	5	5	5
Calcium dihydrogen phosphate	10	10	10	10	10	10	10	10	10	10	10	10	10
Carboxymethyl cellulose	50	49.8	49.2	46.9	37.5	49.8	49.2	46	37.2	49.9	49.4	47.6	40.4
10% astaxanthin ^c^	-	0.20	0.80	3.10	12.5	-	-	-	-	-	-	-	-
10% zeaxanthin ^d^	-	-	-	-	-	0.20	0.80	4.00	12.8	-	-	-	-
10% *β*-carotene ^e^	-	-	-	-	-	-	-	-	-	0.15	0.60	2.40	9.60

The naming convention for the feed is “carotenoid type + carotenoid concentration”. AX, ER, and BC stand for astaxanthin, zeaxanthin, and *β*-carotene, respectively. For instance, “AX-10” indicates that the feed contains 10 mg/kg of astaxanthin. ^a^ Purchased from Xiamen Hailin Biotechnology Co., Ltd. (Xiamen, China); ^b^ purchased from Sichuan CHELOTA Biotechnology Co., Ltd. (Guanghan, China); ^c^ purchased from BASF Co., Ltd. (Beijing, China); ^d^ purchased from Sichuan Anxinlu Biotechnology Co., Ltd. (Xi’an, China); ^e^ purchased from Sichuan Ruiying Biotechnology Co., Ltd. (Xi’an, China).

**Table 2:** Nutrient composition of experimental feeds (%, dry weight, mean ± SD, *n* = 6).

Group	Ash (%)	Moisture (%)	Crude Protein (%)	Crude Lipid (%)	Crude Carbohydrate (%)
Control	10.99 ± 0.68	6.41 ± 0.04	40.15 ± 0.53	3.85 ± 0.18	38.60
AX-10	11.14 ± 0.20	5.50 ± 0.07	40.03 ± 0.13	3.88 ± 0.33	39.45
AX-40	11.65 ± 0.28	4.76 ± 0.05	40.52 ± 0.76	3.45 ± 0.53	39.62
AX-140	11.65 ± 0.16	4.97 ± 0.05	40.52 ± 0.35	3.48 ± 0.20	39.38
AX-500	12.06 ± 0.88	6.26 ± 0.06	39.82 ± 0.08	3.63 ± 0.14	38.23
ER-10	12.09 ± 0.96	6.01 ± 0.05	40.43 ± 0.08	3.99 ± 0.13	37.48
ER-40	12.22 ± 1.18	5.66 ± 0.06	39.75 ± 0.52	3.15 ± 0.15	39.22
ER-140	11.13 ± 0.24	5.08 ± 0.04	40.01 ± 0.08	3.18 ± 0.18	40.60
ER-500	10.24 ± 0.47	7.05 ± 0.04	39.56 ± 0.59	3.22 ± 0.22	39.93
BC-10	10.33 ± 0.47	6.34 ± 0.03	40.74 ± 0.47	2.95 ± 0.04	39.64
BC-40	10.13 ± 0.45	6.28 ± 0.05	40.37 ± 0.41	3.04 ± 0.22	40.18
BC-140	10.59 ± 0.46	6.20 ± 0.00	40.17 ± 0.21	3.03 ± 0.12	40.01
BC-500	10.59 ± 0.49	6.35 ± 0.03	40.14 ± 0.37	3.45 ± 0.34	39.47

The naming convention for the feed is “carotenoid type + carotenoid concentration”. AX, ER, and BC stand for astaxanthin, zeaxanthin, and *β*-carotene, respectively. For instance, “AX-10” indicates that the feed contains 10 mg/kg of astaxanthin.

**Table 3:** The analyzed carotenoid content in diets (mean ± SD, *n* = 6).

	Carotenoid (μg/g)		
	Astaxanthin	Zeaxanthin	*β*-Carotene
Control	-	-	-
AX-10	9.66 ± 0.79	-	-
AX-40	33.9 ± 0.55	-	-
AX-140	123 ± 9.31	-	-
AX-500	467 ± 18.1	-	-
ER-10	-	6.79 ± 0.79	-
ER-40	-	32.9 ± 2.93	-
ER-140	-	147 ± 7.36	-
ER-500	-	522 ± 19.3	-
BC-10	-	-	6.35 ± 1.04
BC-40	-	-	39.7 ± 3.12
BC-140	-	-	139 ± 2.50
BC-500	-	-	530 ± 36.3

The naming convention for the feed is “carotenoid type + carotenoid concentration”. AX, ER, and BC stand for astaxanthin, zeaxanthin, and *β*-carotene, respectively. For instance, “AX-10” indicates that the feed contains 10 mg/kg of astaxanthin.

## Data Availability

Data are contained within the article.

## Supplementary Materials

The following supporting information can be downloaded at: https://www.mdpi.com/article/10.3390/ani14020180/s1, Table S1: biological indices of abalone before thermal and bacterial resistance tests. References [35,36,37] are cited in the supplementary materials.
