# Supplementary material for: Comparative Responses of Orange-Foot and Common-Foot Haliotis gigantea to Carotenoid-Enriched Diets: Survival, Heat Tolerance, and Bacterial Resistance
Source: Animals (Basel). 2024 Jan 5;14(2):180. doi: 10.3390/ani14020180 (PMC10812777; doi:10.3390/ani14020180)
Supplement: Supplementary file 1 [file animals-14-00180-s001.zip › animals-2783011-SI.pdf]

Supporting Information

1. Method and materials

1.1 Composition analysis of compound feeds

The ash content in the feeds was determined with reference to the national standard GB5009.4-2016 and the ash content in the compound feeds was calculated by the equation given below:

The content of ash (%)= $\frac{m_1-m_2-m_0}{m_3-m_2}\times 100$

Where:

m_0 – the mass of magnesium oxide (the product generated from burning of magnesium acetate), in g;

m_1 – the mass of crucible and ash, in g;

m_2 - the mass of crucible, in g;

m_3 - the mass of the crucible and the sample, in g.

Determination of moisture content in feeds: weighed clean and dry glass dish, put into a 100-105 ℃ oven for 10 minutes, cooled down to room temperature, weighed as m_0. Then weighed about 5g of the sample and transferred to the glass dish, with the gross weight of glass dish and the sample denoted as m_1. Placed the dish loaded with compound feeds in a 100-105℃ oven for 2h, cooled down to room temperature, weighed as m_2. The moisture content was calculated by the equation given below:

Moisture content (%) = $\frac{(m_1-m_2)}{(m_1-m_0)}\times 100$

Where:

m_1 – the mass of the crucible and the sample, in g;

m_2 - the mass of the crucible and the sample without moisture, in g;

m_0 – the mass of crucible, in g;

The protein content in feeds was determined with reference to the national standard GB/T6432-2018. The crude protein content was calculated by the equation given below, each sample was determined three times in parallel and the results were averaged as the measured result.

Crude protein content (%) = $\frac{(V2-V1)\times c\times\frac{14}{1000}\times 6.25}{m\times\frac{V'}{V}}\times 100$

Where:

V2 - the volume of hydrochloric acid standard titration solution consumed for titration of the sample (mL).

V1 - the volume of hydrochloric acid standard titration solution consumed by the blank titration (mL).

c - the concentration of standard titration solution of hydrochloric acid (mol/L).

m - the mass of the sample (g).

v - the total volume of the boiling sterilization solution of the sample (mL).

V - the volume of the boiling sterilization solution for distillation (mL).

14 - the molar mass of nitrogen (g/mol).

6.25 - the average coefficient of nitrogen converted to crude protein.

Referencing the national standard GB5009.6-2016, the specific steps are as follows:

The sample, filter paper tube, and cotton thread are placed in an oven at 105°C for 1 hour to completely evaporate the moisture. Then they're quickly cooled to room temperature in a desiccator. The combined weight of the filter paper tube and cotton thread is denoted as m1 (accurate to 0.0001g). Approximately 1g of the sample is loaded into the filter paper tube and securely fastened with cotton thread to prevent leakage. The weight of the filter paper tube containing the sample is denoted as m_2.

The sample-containing filter paper tube is pre-extracted in anhydrous ether for 12 hours. It's then placed in the extraction cylinder of the Soxhlet extractor. The receiving flask is filled with anhydrous ether to a level slightly below the base of the extraction tube. The water bath of the extractor is heated to 80°C, allowing continuous reflux of the ether for 5 hours.

The receiving flask is removed, and the filter paper tube is placed in a fume hood to evaporate most of the ether. It's then placed in a 105°C oven for 1 hour to completely evaporate the remaining ether. It's quickly cooled to room temperature in a desiccator and then weighed.

The fat content in the sample is calculated using the formula:

$$\text{Crude lipid content (\%)} = \frac{m_1 - m_0}{m_2} \times 100$$

Where:

m_1 - initial weight of the filter paper and sample, in g;

m_0 - weight of filter paper and sample after extraction, in g;

m_2 - weight of the sample, in g.

The content of crude carbohydrates in feed is determined using the difference method and is calculated as:

$$\text{Carbohydrates (\%)} = 100 - (\text{Ash (\%)} + \text{Moisture (\%)} + \text{Crude protein (\%)} + \text{Crude lipid (\%)})$$

Table S1. Biological indices of abalone before thermal and bacterial resistance tests

	Shell length (mm)		Weight (g)	
	**Orange foot**	**Common foot**	**Orange foot**	**Common foot**
Control	30.15±2.03	31.06±3.67	3.55±0.55	3.68±0.94
AX-10	31.01±2.82	32.32±2.36	3.49±1.07	3.93±0.85
AX-40	31.35±2.69	30.59±2.63	3.57±1.04	3.49±0.97
AX-140	30.86±2.15	31.78±3.06	3.44±0.77	3.66±1.15
AX-500	30.71±2.29	32.68±2.10	3.37±0.80	4.21±0.74
ER-10	30.56±2.51	31.80±3.15	3.30±0.90	4.16±1.32
ER-40	30.29±2.81	29.98±2.63	3.37±0.92	3.14±0.76
ER-140	30.01±2.98	31.42±2.74	3.41±0.61	3.60±0.88
ER-500	30.92±1.31	31.36±1.70	3.54±0.48	3.59±0.82
BC-10	31.05±3.28	30.19±1.53	3.38±0.93	3.21±0.50
BC-40	30.11±2.09	30.76±1.72	3.03±0.66	3.38±0.66
BC-140	29.74±2.26	31.13±1.46	3.04±0.96	3.59±0.44
BC-500	30.27±2.74	30.47±1.73	3.27±0.99	3.43±0.78

* The naming convention for the feed is "pigment type + pigment concentration." AX, ER, and BC stand for astaxanthin, zeaxanthin, and β-carotene, respectively.

References:

1. GB5009.4-2016. (2016). National food safety standard, determination of fat in foods. Ministry of health of the people's republic of China.

2. GB/T 6432-2018 (2018). Chinese national standard, determination of crude protein in feeds-Kjeldahl method. Ministry of health of the people's republic of China.

3. GB5009.6-2016. (2016). National food safety standard, determination of fat in foods. Ministry of health of the people's republic of China.
